# Soldiers in the Provincial Hospitals

**Published:** 1914-09-19

**Authors:** 


### SOLDIERS IN THE PROVINCIAL HOSPITALS.

(FROM OUR SPECIAL CORRESPONDENTS.)

### First Batch Return to their Depots.

### BRISTOL: DISTINGUISHED VISITORS.

On Thursday, September 10, the Lord Mayor of Bristol paid an official visit to the wounded in the 2nd Southern Hospital, King Edward VII. Memorial Infirmary, Bristol, where, after having been received by the officer commanding—Lieut.-Colonel Paul Bush—he was taken to the operating theatres and afterwards to the various wards, in some of which he addressed the men, and in each case spoke cheery and encouraging words to the wounded. On Friday, last week, the hospital was visited by the Duke of Beaufort, and it is satisfactory to learn that the patients are progressing so well that many have recovered sufficiently to be able to leave for their depôts.

### HARTLEPOOLS HOSPITAL: FROM HOSPITAL TO WORKHOUSE.

Mr. W. C. Gray, J.P., D.L., has offered Tunstall Manor, in conjunction with the Hartlepools and Cameron Hospitals, for wounded officers and men, through Colonel Tuckey. He has also offered Normanhurst for the use of the patients now in the Hartlepools Hospital if occasion should arise for removing them. A special sub-committee, consisting of three members of the medical staff and three of the house committee, was formed in August, when the advisability of closing the hospital was considered, and has now recommended that arrangements be made with the Poor-Law authorities to reserve a ward of twenty-four beds at the workhouse for any patients who may have to be removed from the hospital. Dr. Robertson has been placed in charge of the arrangements for removing the patients, with the assistance of the ambulance corps. The sub-committee's terms of reference on which it submitted the above-mentioned report were "to find suitable quarters for the removal of the patients if necessary, and to make all other necessary arrangements."

### HEREFORD: TERMS WITH THE WAR OFFICE.

The house committee of the General Hospital, Hereford, have offered to place sixty beds at the disposal of the War Office for the use of the sick and wounded, and a communication has been received asking on what terms the hospital is prepared to accept the men. In the meantime certain men recently in camp have received treatment at the hospital, and in response to a request from the War Office authorities for information it was decided to admit any cases at a charge of one guinea a week, which is believed to be the sum charged by other institutions. One suggestion made by Sir Archer Croft is that Herefordshire Territorials invalided home, who need hospital treatment, should receive it free. War Office patients are to be charged 3s. a day, except County Territorials, who will be treated free. Dr. Stanley Lloyd, the house surgeon, has volunteered for active service, and so far, at the time of writing, only a woman doctor has applied for the post. In case of need Colonel H. R. C. Hewat, a member of the board, has offered to take eight of the lighter cases to be treated at his house. It is believed that his example will be generally followed.

### CHATHAM DISTRICT: GERMAN SAILORS.

A properly equipped motor ambulance is being presented to the General Officer commanding the Thames and Medway defences for the easy and rapid conveyance of soldiers who may be brought to the district from the front. The cost is being raised by local subscriptions. Another example of the sympathetic interest which the Royal Family is showing in respect of the wounded was seen last week, when Princess Louis of Battenberg visited Fort Pitt Military Hospital and was conducted round the wards by the officer-in-charge. Her Royal Highness chatted pleasantly with the patients and with some of the wounded German sailors who are also among those under treatment in the institution.

### LEICESTER: MEN RETURN TO DEPOTS.

In his famous History of Leicestershire William Burton, in the reign of James I., wrote that Leicestershire "hath the proportion of an hart, broad at the top and narrower towards the bottom, which shape it truly beareth, for that it lieth almost in the hart and centre of the whole continent of the kingdome. The ayre is generally good, pure, and healthfull, by reason whereof many sweet and pleasant seats and dwellings are here found, healthfull by nature, and much beautified by Art and industry." If this description is true of Leicestershire it is also true of the county town, and in no part of the borough can the air be purer than in the Victoria Park (where is established the military hospital), which stands on an eminence several hundred feet above sea-level, and commands an extensive view of the beautiful Charnwood Forest.

It may be that this purity of air, with its great recuperative qualities, has had something to do with the recovery of the sick and wounded soldiers who were recently received from the seat of war in the 5th Northern General Hospital—in any case it is pleasing to record that after less than a fortnight's stay between thirty and forty soldiers have sufficiently recovered their health to warrant their being sent to their respective depôts. This happy state of things in itself testifies to the fact that the military authorities were right in taking the bold step they did to fit up the old county "asylum" for hospital purposes, and the staff and medical officers are to be congratulated on the effectiveness of their treatment. Further batches of convalescent patients are about to be sent to their depôts, and doubtless their places will be taken, as are the places of those already discharged, by new patients from the military encampments, possibly also from the seat of war. There are at present some 140 patients in the hospital, which has about 400 beds already available for patients. A visit to the hospital shows that substantial progress is being made in the erection of additional buildings with a view to bringing the hospital up to its full complement of 520 beds at an early date.

The hospital has been visited by a number of county residents interested in the treatment of the soldiers, among them the Duke and Duchess of Rutland and their daughter, Lady Diana Manners; Lady Beatty, the wife of Admiral Sir David Beatty, commanding the Battle Cruiser Squadron; Sir Arthur Hazlerigg, Bart., and many others. The thoughtfulness of these among other residents has secured for the men many gifts which the sick and wounded appreciate, such as fruit, warm garments, and, perhaps more prized than all else, pipes, tobacco, cigars, and cigarettes.

### LIVERPOOL: THE BASE HOSPITAL.

A very interesting statement has been submitted by Dr. Utting, chairman of the Port Sanitary Authority and Hospitals' Committee, Liverpool, on the provision which the city is making for the sick and wounded. Early in August the City Infectious Diseases Hospital at Fazakerley was requisitioned by the military authorities as a base hospital. In his capacity as chairman of the Hospitals' Committee Dr. Utting received a deputation from the latter, which included Colonel Edelston, of the West Lancashire Territorial Association, and feeling that he would only be acting on the wishes of the council, he acceded to the request for beds. The hospital provides accommodation for 700, and the colonel-in-charge informed him that there were sufficient stores of all kinds to cater for 1,000 patients if necessary. Already between three and four hundred cases have been received at the institution, consisting not of casualties from the front, but of ordinary sickness from among the Territorials.

Owing to the relative slightness of infectious disease in the city at present, careful arrangement had enabled, without any injustice, the whole staff and patients to be moved to other institutions, so neither the staff employees nor patients have suffered through the change. From the military point of view it is satisfactory to note that General Sir Henry Mackinnon has praised "the splendid building and grounds" placed at his disposal. Moreover, if the need for further accommodation becomes pressing, as a result of an arrangement with the Parks and Gardens Committee, temporary pavilions for tuberculosis cases can be put up on the Harthill estate. An important immediate addition is that of the Liverpool Convalescent Institution, where Alderman Mather, as its chairman, has offered a number of beds for sailors and soldiers when ready to be moved from the base hospital. Owing to the purchase by the Government of 178 horses from the Corporation the Health Committee's recommendation to purchase twenty-four additional motor chassis from a Glasgow firm at a price not exceeding £700 each has been approved.

### OLD PERTH INFIRMARY.

In regard to the steps taken by the directors of the County and City of Perth Royal Infirmary to make available the old infirmary for the War Department—as previously noted in The Hospital—the War Department, to whom the old infirmary had been granted as a base hospital, have asked the Red Cross Society to furnish and equip the buildings to be ready as a hospital. This has now been done, and the building is fully equipped and ready for the reception of wounded soldiers.

### SHEFFIELD: 3rd NORTHERN GENERAL HOSPITAL.

The recovery of sick and wounded who have been receiving attention at the 3rd Northern General Hospital, at Sheffield, since the beginning of the present month has been comparatively quick, as will be appreciated from the fact that of the 125 cases received there have already been discharged, either to duty or on fourteen days' furlough, no fewer than fifty-five. In addition, however, to looking after the sick and wounded, the staff at this base hospital have the important and necessary duties of an invaliding board to discharge, and they have been well occupied in the inevitable weeding-out process to be expected at this stage of the campaign. The men brought before this board have the satisfaction of knowing that their care is in the hands of highly qualified practitioners, for the staff comprises recognised leaders in the various departments of medical science. So well adapted and equipped for the treatment of serious cases is this hospital, and so satisfactory were the detraining arrangements in the case of this first convoy, that it is believed that future convoys to Sheffield will prove of a very much more formidable character. The Sheffield Royal Infirmary and the Sheffield Royal Hospital are also being used in connection with this base—a hundred beds being reserved at the infirmary and forty beds at the hospital. At these institutions the cases requiring operation will be received, thus effecting very considerable economies in the equipment of the general hospital which has been established at the adapted premises of the Sheffield Education Committee's Training College. Another convoy is daily expected.

### TERRITORIALS AT TUNBRIDGE WELLS.

No wounded have as yet reached this area from the front, but the General Hospital has, however, been used by many of the Territorials who are now camped on the Common. Several minor injuries and illnesses are being attended at the out-patient department, and seven have been admitted to the wards for attention. Some eight recruits have had various defects remedied so as to be made fit for service, showing that the voluntary hospital is playing its part at the present time in a quiet way. Very many gifts of linen, blankets, etc., have been received.

**[We hope all Special Correspondents not represented this week will send information in time for our next issue.]**

